# Supplementary material for: Design and Evaluation of Dissolvable Microneedles for Treating Atopic Dermatitis
Source: Pharmaceutics. 2023 Mar 31;15(4):1109. doi: 10.3390/pharmaceutics15041109 (PMC10145410; doi:10.3390/pharmaceutics15041109)
Supplement: Supplementary file 1 [file pharmaceutics-15-01109-s001.zip › pharmaceutics-2271572-supplementary.pdf]

Supporting information

Design and Evaluation of Dissolvable Dexamethasone Microneedles for Treating Atopic Dermatitis

Noa Ben David[a], Yuval Richtman[a], Adi Gross[a], Ruba Ibrahim[b,c] Abraham Nyska[d], Yuval Ramot* [b,c] and Boaz Mizrahi*[a]

[a]Technion- Israel Institute of Technology, Faculty of Biotechnology and Food Engineering, Haifa, 3200003, Israel.

[b]Department of Dermatology, Hadassah Medical Center, Jerusalem, 9112001, Israel

[c]The Faculty of Medicine, Hebrew University of Jerusalem, Jerusalem, 9112001, Israel

[d]Sackler School of Medicine, Tel Aviv University, and Consultant in Toxicologic Pathology, Tel Aviv, 6200515, Israel.

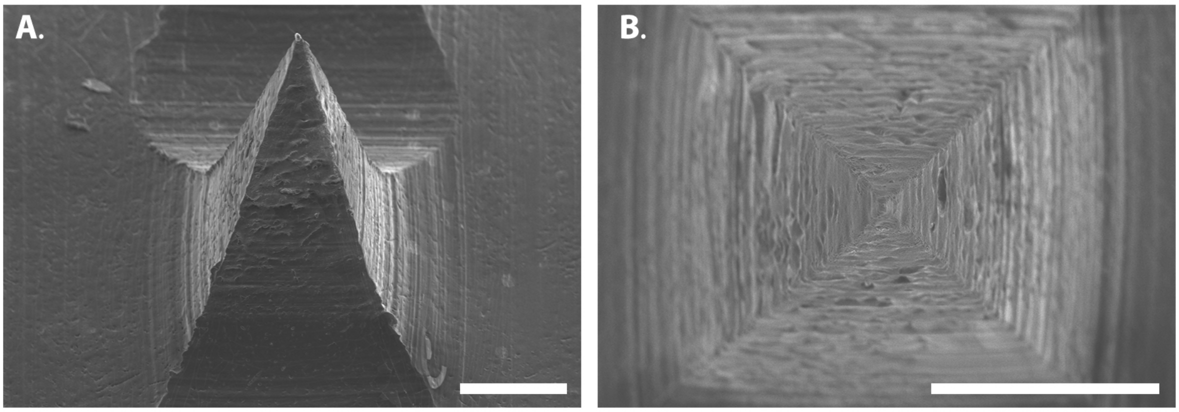

Figure S1. Representative SEM images of well-structured empty MNs Scale bar: 100µm.

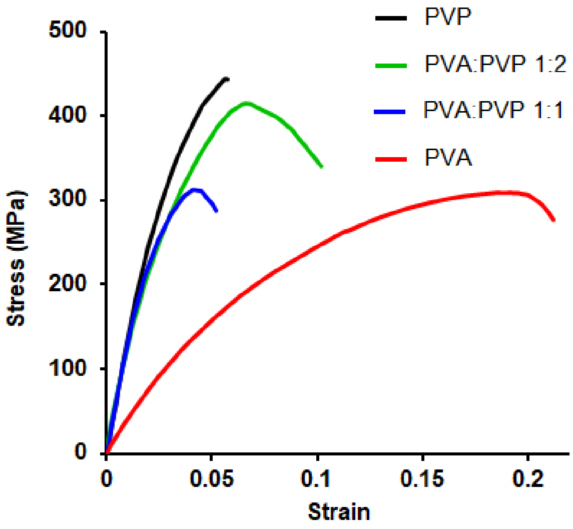

Figure S2. Stress strain behavior of PVA/PVA microneedles in different ratios.

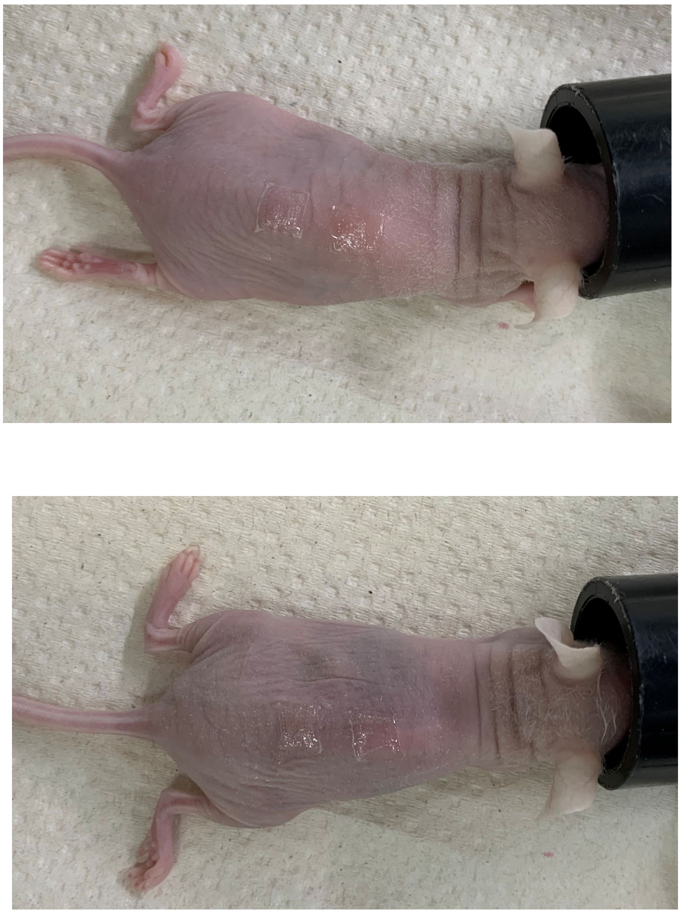

Figure S3. Representative images of MNs 1 h after administration.

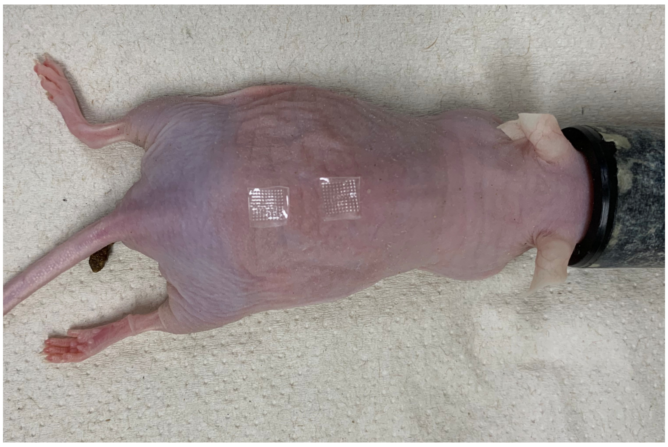

Figure S4. Representative image of MNs placed side by side in the center of the lesion (Not treated, day 13).
